# Targeting sleep quality in acutely traumatized individuals to reduce the risk for PTSD: study protocol for a multicentre randomized clinical trial

**DOI:** 10.1080/20008066.2024.2432163

**Published:** 2024-12-19

**Authors:** Clara Sayk, Jovana Lehmann-Grube, Eva Fassbinder, Sabine Groch, Ingo Schäfer, Ines Wilhelm-Groch

**Affiliations:** a Department of Psychiatry and Psychotherapy, Translational Psychiatry Unit, University of Lübeck, Lübeck, Germany; b Department of Psychiatry and Psychotherapy, Christian-Albrechts Universität zu Kiel, Kiel, Germany; c Department of Psychiatry and Psychotherapy, University Medical Center Hamburg-Eppendorf (UKE), Hamburg, Germany

**Keywords:** Sleep, trauma, PTSD, cognitive behavioural therapy for insomnia, randomized controlled trial, early intervention, mobile health, app, Dormir, trauma, TEPT, terapia cognitive-conductual para el insomnio, ensayo controlado aleatorizado, intervención temprana, salud móvil, aplicación

## Abstract

**Background:** There is a great need for feasible interventions in the initial period after a trauma that minimize the risk of developing a clinically relevant PTSD. The majority of people suffer from severe sleep disorders after a traumatic event. Because sleep is essential for processing emotional memories, we aim to improve sleep quality in acutely traumatized patients to benefit adaptive trauma processing and thereby prevent PTSD development.

**Objective:** In a multicentre randomized controlled trial (RCT), we will investigate whether digital cognitive behavioural therapy for insomnia (dCBT-I) has the potential to not only increase sleep quality in acutely traumatized individuals but also reduce trauma-related symptoms (specific PTSD symptoms as well as related symptoms such as depression, anxiety and functional outcomes) and the risk for PTSD development in acutely traumatized individuals. Moreover, we will test if sleep disturbances pre-therapy predict the development of later PTSD symptoms.

**Method:** We aim to recruit 104 patients who will be randomly assigned to an app-based sleep intervention utilizing CBT-I or a sleep diary as an add-on to treatment-as-usual (TAU) in outpatient acute trauma centres. Sleep quality, PTSD symptoms and everyday functioning will be measured before, after the intervention and at a 6-month follow-up.

**Conclusion:** To our knowledge, this multicentre RCT is the first study to use dCBT-I as an early intervention for trauma. It may improve the understanding of the role of sleep in the development of PTSD and has the potential to contribute to the development of an easy-to-use early intervention for acutely traumatized individuals.

## HIGHLIGHTS

- Sleep disturbances are common after traumatic events and could contribute to the processing of the traumatic event being disturbed.
- This study will increase our understanding of the role of sleep in PTSD development.
- The investigated approach has the potential to open up new possibilities for PTSD prevention.

## Abstract (es)

**Antecedentes:** Existe una gran necesidad de intervenciones factibles en el periodo inicial después de un trauma que minimicen el riesgo de desarrollar un TEPT clínicamente relevante. La mayoría de las personas sufren de trastornos del sueño graves después de un evento traumático. Debido a que dormir es esencial para el procesamiento de los recuerdos emocionales, buscamos mejorar la calidad del sueño en pacientes con trauma agudo para beneficiar el procesamiento del trauma en forma adaptativa y así prevenir el desarrollo del TEPT.

**Objetivo:** En un ensayo controlado aleatorizado multicéntrico (RCT por sus siglas en inglés) investigaremos si la terapia cognitivo conductual digital para el insomnio (dCBT-I por sus siglas en inglés) tiene el potencial de no solo mejorar la calidad del sueño en personas con trauma agudo si no también reducir los síntomas relacionados con el trauma (síntomas específicos de TEPT así como también síntomas relacionados tales como depresión, ansiedad y consecuencias funcionales) y el riesgo de desarrollar TEPT en individuos con trauma agudo. Además, probaremos si los problemas del sueño previos a la terapia predicen el desarrollo de síntomas de TEPT a posteriori.

**Método:** Nuestro objetivo es reclutar 104 pacientes que serán asignados en forma aleatoria a una intervención para el sueño basado en una aplicación utilizando CBT-I o un diario del sueño como un complemento al tratamiento habitual (TAU por sus siglas en inglés) en un centro ambulatorio para pacientes con trauma agudo. La calidad del sueño, los síntomas de TEPT y el funcionamiento diario serán medidos antes, después de la intervención y al seguimiento 6 meses después.

**Conclusiones:** A nuestro entender, este RCT multicéntrico es el primer estudio en usar dCBT-I como una intervención temprana para trauma. Éste puede mejorar la comprensión del papel del sueño en el desarrollo del TEPT y tiene el potencial de contribuir al desarrollo de una intervención temprana fácil de usar para personas con trauma agudo.

## 1. Background

### 1.1. Post-traumatic stress disorder (PTSD) and the need for early interventions

About 50–70% of adults worldwide experience at least one traumatic event in their lifetime, such as war events, physical or sexual violence, accidents or the unexpected death of a loved one. Such traumatic events can cause severe psychological difficulties. The majority of traumatized individuals remits spontaneously. Nevertheless, the conditional risk of PTSD, i.e. the prevalence of PTSD among those exposed to traumatic events ranges from 2.5% (Italy) to 17.6% (Northern Ireland) (Atwoli et al., 2015) with a subgroup of them experiencing the full symptoms of acute stress disorder before the manifestation of PTSD (Bryant et al., 2011). The core symptoms of PTSD encompass re-experiencing the traumatic event in the present (such as intrusions, flashbacks and nightmares), hyperarousal and avoidance of trauma reminders. PTSD and related disorders do not only cause significant distress and impairment of social and daily functioning but may even increase the risk of chronic physical illness (Scott et al., 2013). During the past decades, there has been an increasing effort to develop early psychological interventions aiming to prevent the onset of PTSD. According to the definition from the German guidelines for diagnostics and treatment of acute trauma, all interventions aiming at making the experience more apprehensible and reducing the risk of trauma sequela delivered within the first three months after trauma experience are referred to as early interventions (Bengel et al., 2019). These guidelines recommend early trauma-focused CBT (TF-CBT) for those who exhibit clinical symptoms of post-traumatic stress and active monitoring for those who display subclinical symptom burden (Bengel et al., 2019). However, trauma-focused early interventions are not accessible for the majority of traumatized individuals which increases the risk of manifestation of trauma-related symptoms. In search for feasible early interventions, researchers started to focus on the cognitive and emotional mechanisms that are known to be relevant in the context of PTSD development.

### 1.2. The role of sleep in memory, emotion and PTSD

PTSD can be conceptualized as a memory disorder in which the traumatic event is not properly consolidated and contextualized into autobiographical memory with some aspects of the traumatic event being easily re-experienced after sensory triggers and, oftentimes, amnesia for other parts of the traumatic event (Ehlers & Clark, 2000). According to a review by Colvonen et al. (2019), safety cues are not integrated in the traumatic memory and fear and arousal levels attached to the traumatic memory remain on a high level, leaving the traumatic memory poorly consolidated with incomplete emotional processing.

Healthy sleep enables memory consolidation and contextualization, mostly via transforming labile encoded memory traces into more stable permanent ones through reactivation of the memory during sleep (Rasch & Born, 2013). These memory functions of sleep are mostly related to slow-wave-sleep, more precisely to the coupling of slow oscillations, sleep-spindles and hippocampal ripples during that sleep stage (Born & Wilhelm, 2012). Another function of sleep that is also relevant to the processing of traumatic events is more closely associated with REM sleep. This sleep stage has been implicated in improving fear extinction and emotional processing by reducing the affective load attached to a freshly formed memory (Goldstein & Walker, 2014).

Sleep disturbances feature prominently in the diagnostic criteria for PTSD and Acute Stress Disorder (American Psychiatric Association, 2022). Up to 92% of patients with PTSD suffer from some form of sleep-related symptoms (Milanak et al., 2019). Especially nightmares and insomnia are often reported but restless leg syndrome, sleep apnea and fragmented REM sleep are also widespread (Colvonen et al., 2019) and many sleep disturbances share increased arousal as an underlying factor (Richards et al., 2020). Sleep disturbances are not only common after traumatic events and in PTSD, but pre-traumatic sleep problems and sleep reactivity (Reffi et al., 2023) are also considered a risk-factor for the development of PTSD (Pace-Schott et al., 2023). A spectral index derived from sleep EEG data even is discussed as a potential biomarker for PTSD since it has shown to distinguish patients diagnosed with PTSD from healthy controls with high effect size (de Boer et al., 2019). Both pre- and post-traumatic sleep disturbances might be involved in disturbed processes of fear extinction and emotional processing and a maladaptive consolidation of the traumatic event that are all characteristic of the development and maintenance of PTSD symptoms.

### 1.3. The potential of sleep interventions for trauma processing

As Bryant (2018) states in his review about current findings on of acute stress disorder, early interventions in the aftermath of a traumatic experience should influence key mechanisms that underpin acute traumatic stress. Targeting disturbed sleep in acutely traumatized individuals may provide such a mechanism by improving memory consolidation, emotional processing and fear extinction (Pace-Schott et al., 2023) which already have been identified as important targets for early trauma interventions (Bryant, 2021). Taken together, it can be assumed that sleep-related early interventions have the potential to reduce symptom load and potentially even prevent the development of PTSD (for a detailed discussion of this idea, see also Azza et al., 2020; Larson et al., 2023; Swift et al., 2022).

In our opinion, targeting insomnia symptoms in acutely traumatized individuals has the promise to the most direct route towards altering sleep architecture and spectral activity and thus influence the memory and emotion processing functions of sleep. There are pharmacological as well as psychotherapeutic approaches to target insomnia symptoms. Pharmacological treatment options such as sedative antidepressants, benzodiazepines, antipsychotics and melatonin are limited in applicability since they are either only recommended for short-term use due to risks and side effects, or there is a lack of evidence of their effectiveness (Riemann et al., 2017). Moreover, psychopharmacological agents alter sleep architecture. Benzodiazepines, for instance, decrease the deepest sleep stages as well as REM sleep (de Mendonça et al., 2023) which may hinder memory consolidation processes. Cognitive behavioural therapy for insomnia CBT-I is considered the gold standard and generally is strongly recommended as first-line treatment for chronic insomnia in adults of any age (Riemann et al., 2017).

CBT-I can be conceptualized alongside the cognitive model of insomnia by Morin (1993a) which states that insomnia symptoms are caused by an interaction of maladaptive sleep-related behaviours, cognitions and increased arousal. Accordingly, CBT-I typically includes sleep hygiene, and interventions to improve sleep efficiency such as stimulus control and bedtime restriction, elements of cognitive therapy targeting maladaptive sleep-related beliefs and cognitions as well as relaxation exercises (Harvey & Buysse, 2017). It is not only well established in the treatment of primary insomnia, but also shows considerable effects in comorbid insomnia (Geiger-Brown et al., 2015), including promising trials that used CBT-I as an add-on for the treatment of sleep disturbances in civilian as well as veteran samples meeting PTSD-diagnostic criteria (Colvonen et al., 2019; Talbot et al., 2014). There are also already a few promising studies which have not only examined the impact of CBT-I on sleep disturbances but also on PTSD symptoms in patients suffering from PTSD. Pigeon et al. (2022) reported a medium sized effect of CBT-I on PTSD symptoms. Moreover, in women with probable PTSD a recent study found a clinically relevant reduction of PTSD symptoms in the course of a 5-week manual-based CBT-I (Carlson et al., 2022). However, due to the lack of a control group in this study, it cannot be finally concluded that the reduction in symptoms is a consequence of the intervention and not of spontaneous remission. Gehrman et al. (2020) compared CBT-I delivered via tele-health to an in-person CBT-I program in persons diagnosed with PTSD and did find significant improvement in sleep quality which was comparable in both groups but no effect on PTSD symptoms. These interesting findings show the potential of CBT-I as an intervention for PTSD and raise the question of the effectiveness of CBT-I in acutely traumatized individuals, i.e. before a manifest PTSD has developed. Another advantage of CBT-I is that it can be administered in group interventions, individual therapy and has even become available in a digital format with comparable effects to in-person therapy (Lorenz et al., 2019). The latter also makes it a potentially economical and cost-effective intervention format.

### 1.4. Research questions

We therefore want to investigate whether a digital cognitive behavioural therapy for insomnia (dCBT-I) intervention delivered as an add-on to treatment as usual (TAU) can reduce trauma-related symptoms (specific PTSD symptoms as well as related symptoms such as depression, anxiety and functional outcomes) and the risk to develop PTSD in acutely traumatized individuals. Moreover, in an attempt to replicate previous findings, we will examine whether sleep disturbances pre therapy predict the development of later PTSD symptoms in this heterogeneous sample of acutely traumatized patients. More specifically, in line with previous studies we expect that measures of sleep continuity such as sleep efficiency and nightly awakenings will be especially indicative. They have lately been implicated in predicting PTSD symptoms in an EMA study (Schenker et al., 2023) and could indicate less deep, disturbed sleep and thus worse trauma memory processing (Kleim et al., 2016).

## 2. Methods

### 2.1. Trial design

The planned study is designed as a randomized between-subjects trial with two active groups (one intervention group receiving app-based CBT-I for approximately 8 weeks and one filling in a sleep diary for the same time span). The study intervention functions as an add-on intervention so both groups will receive TAU while undergoing study intervention. Therapists at the study centers delivering the TAU interventions will be blind towards study arm allocation of participants.

### 2.2. Recruitment and setting

**Figure 1.**
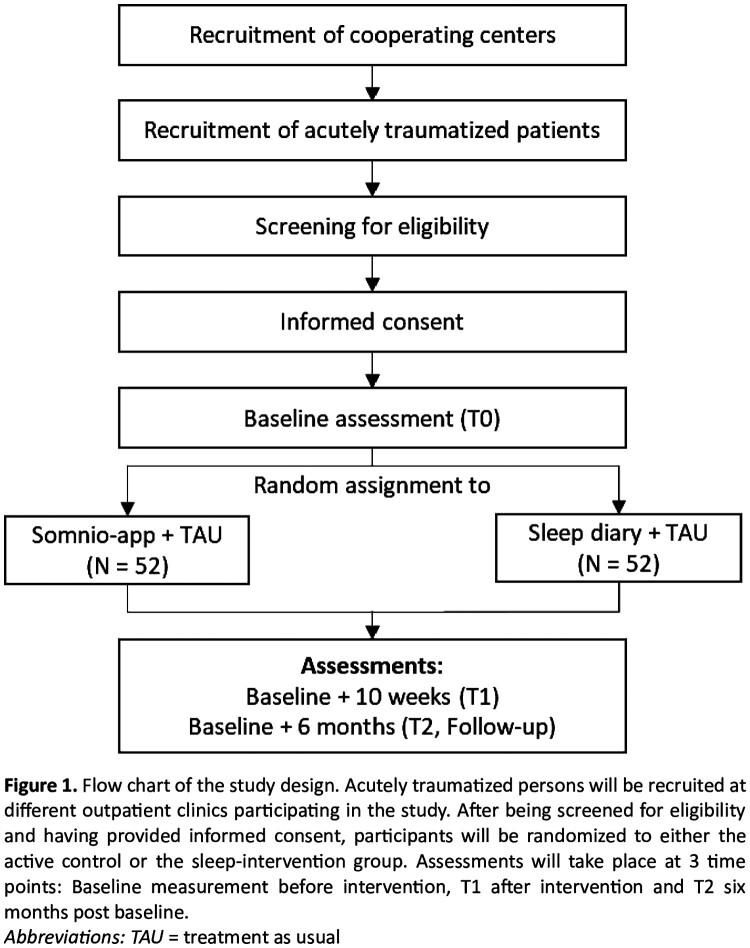
Recruitment process of the study.

Due to probable difficulties of accumulating a large enough sample size in only one center, recruitment is planned as a multicentre study in three outpatient clinics which use similar treatment approaches. To further increase similarity between centres, regular joint supervisions will be utilized. As currently planned, these supervisions will be held by an external supervisor every 6–8 weeks. In addition, we will organize one to two advanced training sessions per year. Therapists of the cooperating clinics will inform potentially eligible patients at the beginning of treatment in oral and written form about the study. If patients give written consent to be contacted by study conductors, clinics will provide patients’ contact data to study conductors. Study conductors will call the respective patients for a screening interview to ensure that all inclusion and exclusion criteria are met and decide on final inclusion in the study. A schematic overview of the whole study procedure is shown in Figure 1.

Cooperations are planned with trauma centers at the Departments of Psychiatry and Psychotherapy at the University of Luebeck as well as at the University Medical Center Hamburg-Eppendorf.

### 2.3. Participants

Participants aged between 18 and 55 years will be included in the study if they (1) have experienced a traumatic event within the past three months as measured with Life Events Checklist (LEC-5; Weathers et al., 2013) and show PTSD symptoms (PTSD Checklist for DSM-5 (PCL-5) score above cut-off value of 33) and (2) report on sleep disturbances which evolved or became stronger after the traumatic experience. Participants will be excluded from the study if they (1) take benzodiazepines or benzodiazepine receptor agonists, (2) report on a lifetime diagnosis of psychosis, bipolar disorder or more than two episodes of major depression, (3) have suffered from an alcohol or substance use disorder within the past year, (4) have an IQ < than 80 points, (5) are diagnosed with any systemic or neurological disorder (e.g. dementia), (6) show signs of acute suicidality, (7) are insufficiently able to communicate in German, (8) work in shifts or have had an intercontinental flight crossing two or more time zones within the past 30 days or (9) suffer from sleep apnea.

### 2.4. Sample size

The planned sample size is based on calculations in G*Power 3.1.9.7 (Faul et al., 2007). Literature investigating the effect of combined sleep- and trauma-focused therapy on trauma-related symptoms is scarce. Pigeon et al. (2022) report a medium sized effect of CBT-I on trauma-related symptoms measured with the Clinician Administered PTSD Scale (CAPS; Blake et al., 1995) in individuals diagnosed with PTSD after having experienced a traumatic event within the past year. Referring to this finding and being interested in a clinically relevant effect, we chose an effect size of Cohen's *f* = .25 as a basis for power analysis and sample size calculation. Calculations setting the desired power to 80% and the risk of Type I error rate to *α* = 5% resulted in a required sample size of *N* = 86 to be able to detect medium sized between factor effects in a mixed design, provided the assumed effects exists in the investigated population. Considering a dropout rate of 20% from guideline-recommended psychological treatments for post-traumatic stress disorder (Varker et al., 2021) as an orientation for potential attrition rate, we intend to recruit *N* = 52 participants per group. Due to the exploratory nature of this pilot study, we used only our primary outcome measure for sample size calculations.

### 2.5. Randomization

Participants will be equally (1:1) and randomly assigned to one of the two between-subjects conditions (intervention group receiving dCBT-I vs. control group filling in a sleep diary every day) while paying attention to matching both groups in age and gender. Study conductors cannot be blind due to practical reasons of study implementation.

### 2.6. Treatments/interventions

Since the intervention is planned as an add-on intervention, all participants will receive TAU in the clinic they are recruited from and are free to use any form of treatment that is offered to them. Due to the multicentre approach, TAU may differ between participants and will be recorded at T1 after the sleep intervention is completed. Potentially different therapeutic approaches in TAU will be included in exploratory data analysis.

#### 2.6.1. Intervention group

Participants in the intervention group will get access to the sleep app *somnio* which serves as the form of dCBT-I employed in this study. *Somnio* is the first digital health application listed permanently with the German Federal Institute for Drugs and Medical Devices for the treatment of insomnia and therefore approved for prescription by medical doctors and psychologists since 2020 (mementor DE GmbH, Leipzig, Deutschland). It belongs to the best rated dCBT-I (Simon et al., 2023) and has been shown to significantly and long-lastingly improve insomnia (Lorenz et al., 2019; Maurer et al., 2023), as well as epiphenomena such as daytime functioning, quality of life, symptoms of depression and anxiety (Schuffelen et al., 2023). Compliance data and completion rates of 68% are promising as well for *somnio*, even in a heterogeneous real-world sample (Schuffelen et al., 2023) which is indeed slightly lower than some reported compliance rates for CBT-I that is delivered in person. However, with 48–91% there is also a wide range of compliance rates in this intervention type (Mellor et al., 2022) and *somnio*'s data fall completely within that range. One activation code is valid for 90 days and users can access the application via app or web browser. The intervention period for this respective study is planned for approximately 8 weeks. The intervention program consists of 10 educative core- and 6 concomitant-modules which are based on Morins microanalytic model of insomnia (Morin, 1993a). In this model, it is stated that insomnia is primarily sustained by a cycle of self-perpetuating factors such as high arousal, negative consequences, maladaptive habits and dysfunctional cognitions about sleep (Perlis et al., 2005). Users are guided through the intervention program by an animated sleep coach called Albert to create a tailored dialogue that is based on pre-programmed input by CBT-I experienced psychotherapists. A list and description of all educative modules and the closing session can be found in Table 1.

**Table 1.** Content of dCBT-I application somnio.

Module	Content description
Introduction	Introduction to therapy content and procedure, personalization of settings, recording of sleep symptoms and therapy goals
Sleep diary	Introduction to sleep diary as basis for subsequent personalized training and instructions on how to fill in the reports
Sleep knowledge	Psychoeducation on sleep phases, 2-process model of sleep (sleep pressure and circadian rhythm) and individual differences concerning sleep and chronotypes
Practical exercise	Deepening of sleep-related knowledge using practical exercises (taking the role of a sleep expert who gives advice to a patient suffering from insomnia similar to the user's symptoms)
Cycle of insomnia	Introduction to cycle of insomnia according to cognitive model of insomnia (Morin, 1993a) including dysfunctional sleep behaviours, hyperarousal, dysfunctional cognitions, and consequences; creation of individual insomnia model
Sleeping hours	Sleep time, introduction of sleep restriction and definition of individual sleep window (based on individual mean sleep duration within the last 7 days reported in the sleep diary)
Relaxation	Relaxation, introduction of progressive muscle relaxation and instruction to practice further with a provided audio file (aim of reducing physical and mental tension to enhance sleep onset latency and waking after sleep onset)
Sleep behaviour	Sleep behaviour, improvement of dysfunctional sleep habits (sleep hygiene) and introduction of stimulus control principle
Thoughts	Sleep-related cognitions, introduction of connection between emotions and cognitions and consequences of dysfunctional sleep-related cognitions; instruction to identify and dispute dysfunctional sleep-related cognitions
Everyday decisions	Integration of therapy content into everyday decisions by presenting different scenarios for which the user has to decide on how to behave
Closing session	Brief repetition of therapy content and quiz, reflection on goals and achievements, planning of time succeeding the intervention program

Note. dCBT-I, digital cognitive-behavioural therapy for insomnia.

#### 2.6.2. Control group

The control group will be asked to fill in a sleep diary in online format (link will be sent by email everyday) for 8 weeks which is the same time span as the *somnio* app is processed by the intervention group. For ethical reasons, we intend to offer a *somnio* app activation code to participants of the control group after study completion, too.

### 2.7. Procedure and assessment

After the recruitment procedure is completed, participants will provide written informed consent and are randomized to one of the two study conditions. Participants will then receive a link to an online questionnaire arranged in SoSciSurvey for baseline assessment (timepoint T0). Following baseline assessment, the intervention group will get access to the *somnio* app and receive dCBT-I for the subsequent 8 weeks whereas the control group will fill in an online sleep diary every day (link provided by email). Five weeks after the start of the intervention, participants will receive a phone call to encourage compliance and spot potential issues with the use of the app or sleep diary. Both groups will continue to participate in TAU of the respective clinic without any limitations. After the intervention phase (10 weeks post baseline), all participants will receive a link to a second online assessment (timepoint T1). Additionally, the researchers conducting the study will contact every participant for a phone interview around T1 to gather information about current medication, number, and content of trauma-related outpatient treatment sessions. Six months after baseline assessment, participants will be contacted again and asked to fill in a follow-up assessment (T2). All measures used for the assessments on different time points are listed in Table 2. At all assessments, participants have the possibility to pause filling out the questionnaires in case they become overwhelmed and are allowed to resume at a later point during assessment day.

**Table 2.** Measures used for the assessments on different time points.

	Pre-intervention	Post-intervention	
	T0	T1	T2
Demographic data	x		
Sleep diary	x	x	x
MCTQ	x		
PSQI	x	x	x
LEC-5	x		
PCL-5	x	x	x
PCTI	x	x	x
CTQ	x		
BDI-II	x	x	x
BAI	x	x	x
WHODAS 2.0	x	x	x

Abbreviations: *BAI*, Beck Anxiety Inventory; *BDI-II*, Beck-Depressions-Inventar; *CTQ*, Child Trauma Questionnaire; *LEC-5*, Life Events Checklist for DSM-5; *MCTQ*, Munich ChronoType Questionnaire; *PCL-5*, PTSD Checklist for DSM-5; *PCTI*, Post-traumatic Cognitions Inventory; *PSQI*, Pittsburgh Sleep Quality Index; *WHODAS 2.0*, WHO Disability Assessment Schedule.

x, Instrument is assessed at respective timepoint.

### 2.8. Outcome measures

#### 2.8.1. Primary outcome measures

The primary outcome measures are PTSD symptom severity and number of participants with probable PTSD according to a PCL-5 cut-off of 33, which showed the best sensitivity-to-specificity-ratio for detecting PTSD (Weathers et al., 2013). Due to the online-design, we could not use CAPS interview as Pigeon et al. (2022) did to survey PTSD symptom development and decided to use PTSD Checklist for DSM-5 (PCL-5; Weathers et al., 2013) and Post-traumatic Cognitions Inventory (PCTI; Foa et al., 1999) as self-reports instead, both applied at T0, T1 and T2. Whereas the PCL-5 covers all PTSD symptoms, PTCI specifically measures trauma-related cognitions. The PCL-5 asks respondents to rate bothering of PTSD symptoms during the past month on a 5-point likert scale (1 = not at all to 5 = extremely) and has strong internal consistency (*α* = .94), test–retest reliability (*r* = .82), and convergent (*rs* = .74 to .85) and discriminant (*rs* = .31 to .60) validity (Blevins et al., 2015). The PTCI consists of 33 questions about trauma-related cognitions (based on the three factors Negative Cognitions About Self, Negative Cognitions About the World and Self-blame) which respondents rate on a 7-point-likert scale (absolutely no consent to strong consent). As PCL-5, PTCI shows excellent psychometric properties with internal consistency ranging from *α* = of .86–.97 (Foa et al., 1999). As the PCL-5 contains the item ‘Trouble falling or staying asleep?’ (Weathers et al., 2013), all analyses for the PCL-5 will be conducted with and without this item separately to detect whether potential changes on this measurement are solely driven by changes in sleep quality.

#### 2.8.2. Secondary outcome measures

Secondary outcome measures are (1) sleep quality measured by sleep diary entries (*Schlaffragebogen A*, SF-A/R; Görtelmeyer, 2011) filled in by both groups for 1 week around every time point, the insomnia severity index (ISI; Morin, 1993b), and the Pittsburgh Sleep Quality Index (PSQI; Buysse et al., 1989) including the addendum for PTSD (PSQI-A; Germain et al., 2005), a broader measure of sleep quality, that does not only include insomnia symptoms but also other symptoms that can occur in traumatized individuals, such as bad dreams and breathing problems during sleep, (2) depressive symptoms measured with the Beck Depression Inventory (BDI-II; Beck et al., 1996), (3) anxiety assessed with the Beck Anxiety Inventory (BAI; Beck et al., 1988) and (4) general functioning in everyday life quantified by the WHO Disability Assessment Schedule (WHODAS 2.0; Üstün et al., 2010). Sleep diary entries for measurement of sleep quality are additional and independent from the sleep diary included in the *somnio* program in the intervention group and the 8-week sleep diary planned as intervention for the control group. For pre and post measurement they are administered in the week before the intervention and the week after the intervention respectively to avoid any overlap with the sleep diaries during the intervention period.

All measures have shown to have good psychometric properties. For the PSQI, studies indicate high internal consistency (*α* = .75–.80) as well as moderate to high construct validity (Carpenter & Andrykowski, 1998; Hinz et al., 2017). For the BDI-II, internal consistency is described as around .9, the retest reliability ranges from .73 to .96 and it shows good sensitivity and specificity in detecting depression (Wang & Gorenstein, 2013). The BAI has high internal consistency (*α* = .92) and a test–retest reliability of *r*(81) = .75 over 1 week and discriminates well among different anxious groups (Beck et al., 1988). For the WHODAS 2.0, studies indicate high internal consistency ranging from *α* = .70–.97 in different populations and support reliability, validity, dimensionality and responsiveness of its German version (Pösl et al., 2007).

### 2.9. Statistical analysis

Statistical analyses will be based on a mixed general linear model design with the two-level between group factor intervention (intervention vs. control group) and the three-level within group factor time (assessments at T0, T1 and T2) for primary and secondary outcome measures to account for both hypotheses tested in this study. A multilevel approach including study centers with their, presumably, differing TAU interventions, is not feasible due to too low a number of study centers at this hierarchical level. However, as discussed below, study centre and TAU interventions will be used as covariates and for exploratory analyses. For the first research question, the interaction between groups and time will be analysed to determine whether PTSD symptom severity differs for the intervention group after receiving the add-on treatment. For the second research questions, sleep disturbance before the intervention will be used to predict PTSD symptom severity at first and second follow-up for both groups. Due to the exploratory nature of this study, analysis will be conducted both with an intention-to-treat and a per-protocol approach, allowing for a comparison of both effect estimates (Gupta, 2011). For intention-to-treat analysis, dropouts will be included with a last observation carried forward strategy. Extreme cases and outliers will be checked for validity and excluded for per-protocol analysis if indicated. Dropouts will be reported with time point and reason in the final results. We intend to include age, gender, severity of trauma (indicated by classification in type-1 and -2 traumata), number of previous traumatic events, content of trauma-related outpatient treatment sessions and recruiting centre as covariates and in further exploratory analyses. Despite our efforts to proactively reduce variance in trauma treatment among participating trauma centers by organizing joint supervisions, we assume there will be left some significant variance that has to be considered in the statistical analyses. The content of trauma-related outpatient treatment sessions will be classified into trauma-focused versus non-trauma-focused therapy elements since it has been shown that in symptomatic acutely traumatized individuals this may cause a significant difference in treatment outcome (Roberts et al., 2019). Furthermore, we will survey whether sleep interventions have been included in the therapy process or not. By this, we intend to control for the most relevant confounding factors arising from between-participant-treatment-differences.

## 3. Discussion

In this study protocol, we describe an RCT trial using a CBT-I intervention delivered via app as an add-on to TAU in a multicentre study. It is designed to explore (i) if an early sleep intervention can reduce sleep disturbances and trauma-related symptoms in patients with acute trauma and (ii) if severity of sleep disturbances before the intervention predicts the development of PTSD symptoms later on. The choice of using an RCT design with the intervention being an add-on to TAU strikes the balance between methodological rigor and the explorative nature of this study wanting to ensure patients receive the treatment they individually need in a naturalistic setting. As far as the authors know, this is the first trial using this type of intervention in acutely traumatized individuals. It has the potential to shed light on the role of sleep and sleep disturbances in acute trauma and may provide a new and easily to administer early intervention. Thereby, it covers an approach that has been suggested in recent review articles (Azza et al., 2020; Swift et al., 2022).

We believe this study has several strengths. A major one is the strong theoretical framework connecting the cognitive and emotional processes after a traumatic event, sleep and CBT-I. With the early implementation of CBT-I the memory consolidation and emotional processing properties of healthy sleep can be utilized at a time in post-traumatic symptom development, when maladaptive processing of the traumatic event might have already started but is not yet manifested. Another advantage of the early intervention is, that it has the potential to prevent not only manifestation of trauma-related symptoms and the associated suffering, but it could even help to save costs for healthcare and workdays lost.

One issue, however, inherent to the exploratory nature of the study, is that it is yet unknown, how the specific population of acutely traumatized individuals will react to the CBT-I intervention. There might be unexpected side effects or increased adversity of known CBT-I side effects such as initially increased tiredness during daytime after sleep restriction (Kyle et al., 2011). However, studies using sleep interventions in patients with chronic PTSD do not support this notion (Colvonen et al., 2019; Talbot et al., 2014). Importantly, such side effects will be monitored at T1, patients continue to be seen at the acute trauma clinics and can always seek any additional psychotherapeutic or pharmacological assistance (except for benzodiazepines) that they might need within study protocol. Additionally, the study team is available in case participants have any questions or wish to report adverse events.

There also are some considerations arising from balancing the methodological rigor and the explorative nature of this study. As mentioned before, TAU is likely going to vary across study centers, therapists and a heterogeneous population of patients and therefore needs to be controlled for both by randomization as well as assessment and later use of covariates. While this approach does indeed introduce more variance than in a completely controlled study protocol, it can also be considered a strength of this explorative study. The ‘real-world’ context actually provides a much stronger trial for a novel intervention and will render any potential effects of the CBT-I intervention more meaningful. It might even indicate which other interventions CBT-I is best paired with. The issue of using a multicentre approach in this study can be reflected upon with a similar argument. It will assure wider (e.g. geographical) participation, and therefore greater generalizability and representativeness of results.

Apart from considerations around the study design, there are some methodological limitations. First, the sleep intervention being delivered in digital format forces the participants to process the intervention content self-reliantly. This may lead to decreased compliance compared to in-person interventions, where therapists can directly address compliance. As we have access to app use and sleep diary data however, we are able to measure participation in the intervention and control group. Second, and related to patient compliance is the self-selection of more motivated and possibly also less severely impaired patients in the CBT-I group. To take this into account we will conduct an intent-to-treat analysis, and we will analyse characteristics of all dropouts to detect potential differences between both groups. Third, beside of sleep questionnaires indicating subjective sleep quality we decided to not include any objective sleep measurements. Thus, changes in sleep architecture that might be related to potential changes in trauma processing cannot be detected. For this first exploratory study, it is more important for us to include a high number of patients at different centres within a reasonable period of time to uncover possible effects of CBT-I at the behavioural level. If this approach is successful, objective sleep data should also be collected in subsequent studies to investigate underlying mechanisms.

## 4. Conclusion

Sleep and sleep disturbances are likely to be an important factor in the development and maintenance but also in the treatment and prevention of PTSD due to the memory and emotional functions of sleep. This multicentre RCT could be the first trial of idea to use CBT-I as an early intervention in traumatized individuals and has promise to increase understanding of sleep’s role in PTSD development as well as providing new avenue for easy to administer early intervention.

The publication of this study protocol is not only in line with the principles of open science, but it is also intended to accelerate further research in what we consider a very innovative field, especially given that data collection and, consequently, the publication of such clinical studies often take a very long time.

## Data Availability

Data sharing is not applicable to this article as no new data were created or analysed.
